# Salinomycin: Anti-tumor activity in a pre-clinical colorectal cancer model

**DOI:** 10.1371/journal.pone.0211916

**Published:** 2019-02-14

**Authors:** Johannes Klose, Stefan Trefz, Tobias Wagner, Luca Steffen, Arsalie Preißendörfer Charrier, Praveen Radhakrishnan, Claudia Volz, Thomas Schmidt, Alexis Ulrich, Sebastian M. Dieter, Claudia Ball, Hanno Glimm, Martin Schneider

**Affiliations:** 1 Department of General, Visceral and Transplantation Surgery, University of Heidelberg, Heidelberg, Germany; 2 Translational Functional Cancer Genomics, National Center for Tumor Diseases (NCT) Heidelberg and German Cancer Research Center (DKFZ), Heidelberg, Germany; 3 Department of Translational Medical Oncology, National Center for Tumor Diseases (NCT) Dresden and German Cancer Research Center (DKFZ), Dresden, Germany; 4 Center for Personalized Oncology, University Hospital Carl Gustav Carus Dresden at TU Dresden, Dresden, Germany; 5 German Consortium for Translational Cancer Research (DKTK) Dresden, Dresden, Germany; University of Pécs Medical School, HUNGARY

## Abstract

**Objectives:**

Salinomycin is a polyether antibiotic with selective activity against human cancer stem cells. The impact of salinomycin on patient-derived primary human colorectal cancer cells has not been investigated so far. Thus, here we aimed to investigate the activity of salinomycin against tumor initiating cells isolated from patients with colorectal cancer.

**Methods:**

Primary tumor-initiating cells (TIC) isolated from human patients with colorectal liver metastases or from human primary colon carcinoma were exposed to salinomycin and compared to treatment with 5-FU and oxaliplatin. TICs were injected subcutaneously into NOD/SCID mice to induce a patient-derived mouse xenograft model of colorectal cancer. Animals were treated either with salinomycin, FOLFOX regimen, or salinomycin and FOLFOX. Human colorectal cancer cells were used to delineate an underlying molecular mechanism of salinomycin in this tumor entity.

**Results:**

Applying TICs isolated from human patients with colorectal liver metastases or from human primary colon carcinoma, we demonstrated that salinomycin exerts increased antiproliferative activity compared to 5-fluorouracil and oxaliplatin treatment. Consistently, salinomycin alone or in combination with FOLFOX exerts superior antitumor activity compared to FOLFOX therapy in a patient-derived mouse xenograft model of colorectal cancer. Salinomycin induces apoptosis of human colorectal cancer cells, accompanied by accumulation of dysfunctional mitochondria and reactive oxygen species. These effects are associated with expressional down-regulation of superoxide dismutase-1 (SOD1) in response to salinomycin treatment.

**Conclusion:**

Collectively, the results of this pre-clinical study indicate that salinomycin alone or in combination with 5-fluorouracil and oxaliplatin exerts increased antitumoral activity compared to common chemotherapy.

## Introduction

Colorectal cancer is one of the most common malignancies worldwide with the fourth highest prevalence among females and males and a lifetime risk of 1 in 20 persons [1,2]. While the combination of radical surgical resection and neo- and/or adjuvant (radio)chemotherapy results in 5-year survival rates of 65%, metastasized colorectal cancer is associated with decreased long-time survival [3]. Colorectal liver metastases are the most common cancer-related cause of death, leading to 5-year survival rates of less than 15% [2]. Chemotherapy is fluoropyrimidine-based combined with oxaliplatin or irinotecan and monoclonal antibodies targeting vascular endothelial growth factor (VEGF) or, in case of no *KRAS* mutations, endothelial growth factor receptor (EGFR). Effective chemotherapy in the metastasized or palliative situation is often hindered due to a small fraction of phenotypically different cells within the primary tumor, which exhibits an increased tumorigenic potential and retains resistance against chemotherapy and formation of metastases. This subpopulation of cells is commonly referred to as cancer stem cells [4]. Until today, no specific anti-cancer stem cell therapy exists.

Salinomycin was shown to exhibit selective inhibitory effects against human breast cancer stem cells [5]. Consequently, activity of salinomycin has been confirmed in numerous types of cancer, including non-solid malignancies [6], brain [7], bone [8], and lung cancer [9], as well as gastrointestinal tumors [10–13]. The activity of salinomycin against colorectal cancer cells has been demonstrated *in vitro* and *in vivo* before applying immortalized cell lines [14–16].

Before the initiation of clinical studies, the effectiveness of salinomycin in primary human colorectal cancer cells has to be demonstrated. For this purpose, tumor-initiating cells (TIC) represent an attractive source to prove the activity of novel cancer drugs [17]. Isolation and characterization of TICs derived from human colorectal cancer tissue have been described in detail before [18–20]. In this pre-clinical experimental study, we investigated the anti-cancer activity of salinomycin in three TIC cultures derived from colorectal liver metastases and one TIC culture from a primary colon cancer. The effectiveness of salinomycin was compared to treatment with a combination of 5-fluorouracil, folic acid, and oxaliplatin, commonly referred to as FOLFOX regimen. We show in several in vitro-assays that salinomycin exerts anti-stem cell activity against all four TIC cultures, where FOLFOX therapy exerts only weak effectiveness. In NOD/SCID mice, patient-derived subcutaneous xenograft models were conducted. Tumor growth was likewise inhibited by salinomycin treatment.

## Results

### Exposure to salinomycin reduces the viability of TIC

**Fig 1:**
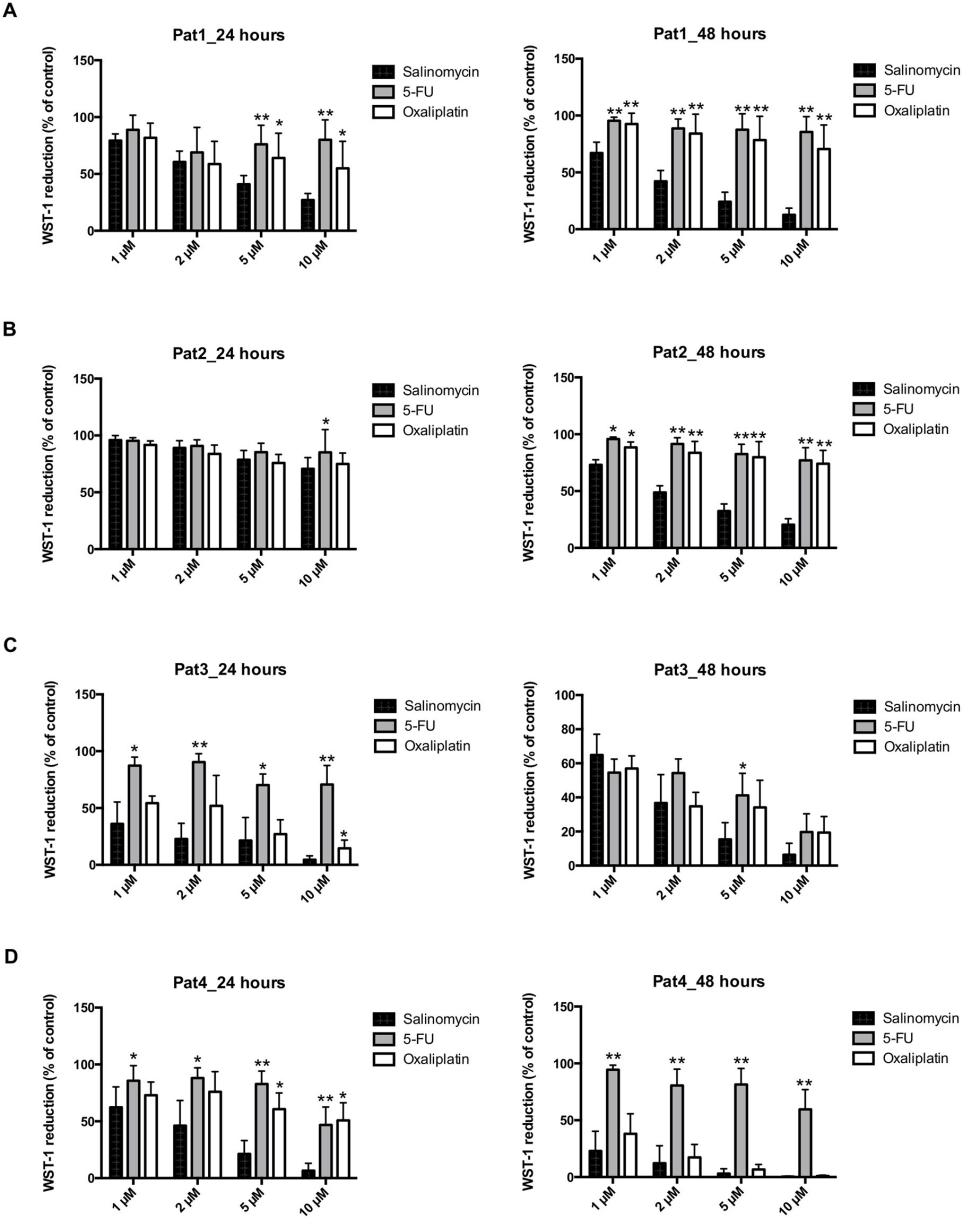
Salinomycin reduces the cell number of colorectal cancer TICs. TIC cultures from patients 1–4 were cultured in the absence or presence of increasing concentrations of salinomycin, 5-fluorouracil, and oxaliplatin (1, 2, 5, and 10 μM) for 24 and 48 hours. Tumor cell number was analyzed by WST-1 reduction, which is assumed to be proportional to the cancer cell number. Results are shown as summary of n = 4 independent experiments as mean ± SEM. * p < 0.05, ** p < 0.001 compared with salinomycin treatment.

First, we analyzed the activity of salinomycin in three TIC lines derived from colorectal liver metastases (Pat 1–3) and one TIC line from colon cancer (Pat 4) using the WST-1 assay. Each spheroid culture was exposed to increasing concentrations of salinomycin, 5-FU, and oxaliplatin at equivalent dosages (1, 2, 5, and 10 μM) for 24 and 48 hours. As demonstrated in Fig 1A–1D, salinomycin significantly and dose-dependently reduced tumor cell viability in all spheroid cultures after 24 and 48 hours of treatment compared to 5-FU. Treatment with oxaliplatin also partially (patients 3 and 4) resulted in reduced TIC viability.

To further corroborate these findings, we applied the CellTiter-Glo Assay to assess the viability of TICs derived from colorectal liver metastases or primary colon carcinoma. Cell viability of all four spheroid cultures was consistently reduced after exposure to increasing concentrations of salinomycin (1, 2, 5, and 10 μM) for 24 and 48 hours (S1A–S1D Fig). Salinomycin treatment exhibited superior activity against TIC compared to treatment with 5-FU or oxaliplatin. Of note, exposure of TICs from patient 3 and patient 4 to oxaliplatin also resulted in decreased cell viability, as already observed in the WST-1 assay.

### Salinomycin induces apoptotic death of TICs

**Fig 2:**
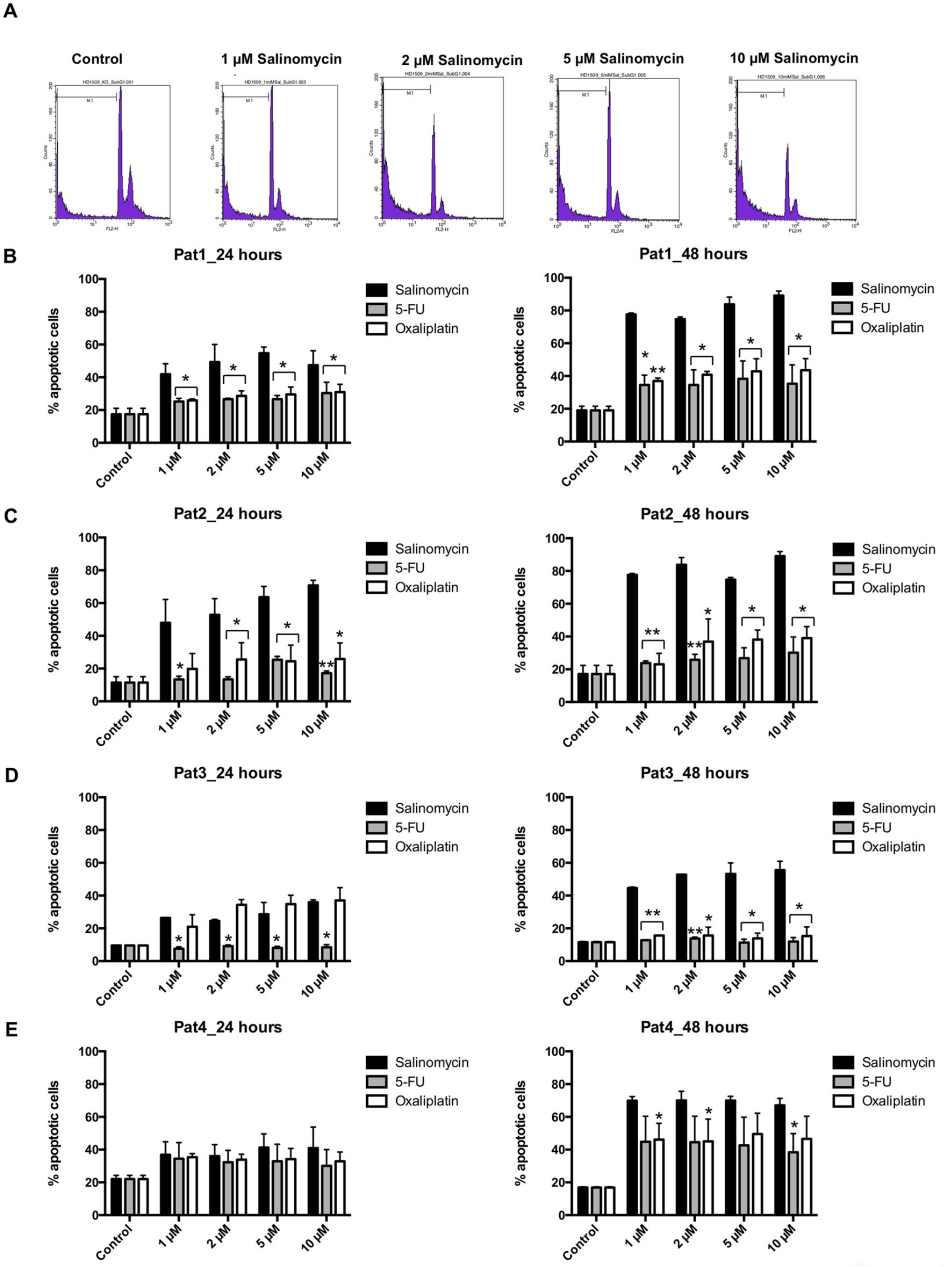
Salinomycin induces apoptosis in colorectal cancer TICs. TIC cultures from patients 1–4 were cultured in the absence or presence of increasing concentrations of salinomycin, 5-fluorouracil, and oxaliplatin (1, 2, 5, and 10 μM) for 24 and 48 hours. Induction of apoptotic cell death upon treatment was performed by SubG1 analysis. Data are shown as representative histograms of flow-cytometric analysis with a logarithmic (A) or linear (S2A Fig) amplification of DNA fluorescence or as summary of n = 3 independent experiments as mean ± SEM (B-E). * p < 0.05, ** p < 0.001 compared with salinomycin treatment.

To analyze the induction of cell death in TICs isolated from colorectal liver metastases or primary colon cancer, we examined DNA fragmentation after exposure to salinomycin, or to 5-FU and oxaliplatin (1, 2, 5, and 10 μM) for 24 and 48 hours. The sub G1 population was regarded as apoptotic cell fraction [21]. A representative histogram of flow-cytometry analysis is depicted in Fig 2A. As demonstrated in Fig 2B–2E, treatment with salinomycin induced apoptotic cell death in all spheroid cultures used in this study. Strong pro-apoptotic activity of salinomycin was observed in TICs derived from patients 1–3. Treatment with 5-FU and oxaliplatin also induced apoptosis in TICs from patients 1 and 2 (Fig 2B and 2C). Oxaliplatin induced apoptosis in TICs derived from patient 3 after 24 hours as well. In TICs obtained from patient 4, treatment with 5-FU and oxaliplatin induced apoptosis comparable to salinomycin (Fig 2E).

Induction of apoptosis in TICs was also analyzed applying AnnexinV analysis. As shown in S2B Fig, treatment with salinomycin resulted in induction of apoptosis in a dose-dependent manner. Consistently with the results obtained in detection of the sub G1 population, induction of apoptosis was observed after treatment with salinomycin, 5-FU, or oxaliplatin in all patient-derived TICs (S2B Fig).

### Anti-stem cell activity of salinomycin in TIC cultures

**Fig 3:**
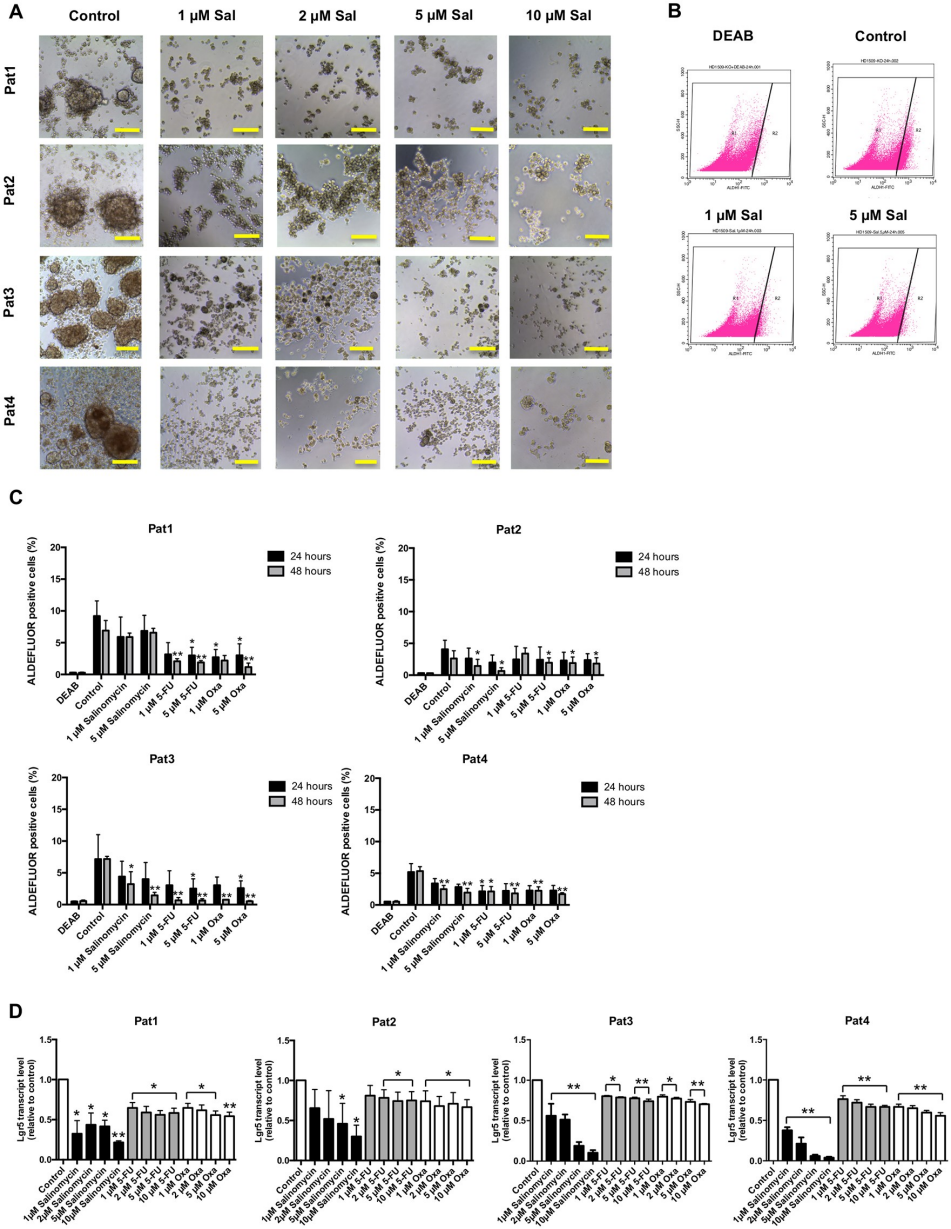
Anti-stem-cell activity of salinomycin in colorectal cancer TICs. (A) TIC cultures from patients 1–4 were cultured in the absence or presence of increasing concentrations of salinomycin (1, 2, 5, and 10 μM) for 21 days. Cell morphology and sphere formation capacity was assessed daily and cell cultures were documented after end of treatment. Results are shown as representative images (n = 3 individual experiments) of treated TIC with salinomycin (B + C). The activity against cancer stem cells was further analyzed by measurement of the ALDH1^+^ population after treatment for 24 and 48 hours. Results are displayed as representative dot blots (B) or as summary of n = 3 independent experiments as mean ± SEM (C). Analysis of mRNA expression level of Lgr5 after treatment with increasing concentrations of salinomycin, 5-FU, and oxaliplatin for 24 hours was further investigated and shown as summary of n = 3 independent experiments as mean ± SEM (D); * p < 0.05 and ** p < 0.001 compared with salinomycin treatment. Scale bars = 100 μM.

Spheroid formation of TICs is regarded as a hallmark of tumorigenic capability and obligatory to form de novo tumors in immunocompromised mice [17]. To assess the impact of salinomycin on spheroid formation, we exposed all four TIC lines to increasing concentrations of salinomycin (1, 2, 5, and 10 μM) for 21 days. Strikingly, salinomycin dose-independently inhibited spheroid formation in all four patient-derived TIC cultures (Fig 3A). In comparison, after treatment with 5-FU or oxaliplatin, spheroid formation was still detectable even after exposure to high drug concentrations after three weeks of treatment (S3 and S4 Figs).

Stem cell surface markers like CD133, CD44, or EpCam have been used to enrich colorectal cancer TICs and to reflect their tumor-initiating properties [22]. Therefore, we investigated the impact of exposure of TICs to salinomycin (1, 2, 5, and 10 μM) for 24 hours on the surface expression of CD133, CD44, and EpCam. As demonstrated in S5 Fig, stem cell marker expression was heterogeneous among the four TIC cultures. While EpCam was consistently expressed in all cell lines, a high CD133 expression was only observed in TICs derived from patient 1. TICs from patient 2 revealed only moderate CD133 expression while it was absent in TICs from patients 3 and 4. CD44 was moderately expressed in all cell lines. Exposure with salinomycin did not alter the stem cell marker expression pattern in all four TIC cultures. Given that the expression of stem cell surface markers is not predictive for the proliferation or tumor initiating capacity of the TIC cultures used in this study [19], we did not analyze the impact of 5-FU and oxaliplatin on the stem cell marker expression.

Aldehyde dehydrogenase-1 (ALDH1) is regarded as a marker to label colorectal cancer stem cells [23]. Its relevance for the tumorigenity of the TIC cultures used in this study has not been investigated so far. Therefore, we assessed ALDH1 expression after treatment with salinomycin, 5-FU, and oxaliplatin for 24 and 48 hours. Exposure of all TIC lines to salinomycin resulted in reduction of the ALDH1^+^ population, particularly after treatment for 48 hours. Of note, ALDH1^+^ reduction was less pronounced in TICs derived from patient 1 (Fig 3B+3C). Strikingly, exposure to 5-FU and oxaliplatin resulted in a more pronounced reduction of the ALDH1^+^ population compared to salinomycin treatment. This effect was observed in all four TIC cultures (Fig 3C).

We further investigated the anti-stem cell activity of salinomycin, 5-FU, and oxaliplatin in patient-derived TICs applying qPCR to analyze the mRNA expression of Lgr5, which is regarded as substantial contribution to the colorectal cancer stem cell hierarchy and to colorectal carcinogenesis [24]. As demonstrated in Fig 3D, salinomycin-exposure for 24 hours resulted in a dose-dependent reduction of Lgr5 mRNA expression in all four TIC lines. Exposure to increasing concentrations of 5-FU or oxaliplatin did also alter Lgr5 mRNA expression in TICs. Of note, the suppressive effect on Lgr5 mRNA expression was more pronounced after treatment with salinomycin in comparison to common chemotherapeuticals. TICs derived from patient 1 did not react in the same manner compared to TICs derived from patients 2–4 (Fig 3D).

### Salinomycin inhibits tumor growth in a patient-derived xenograft model

**Fig 4:**
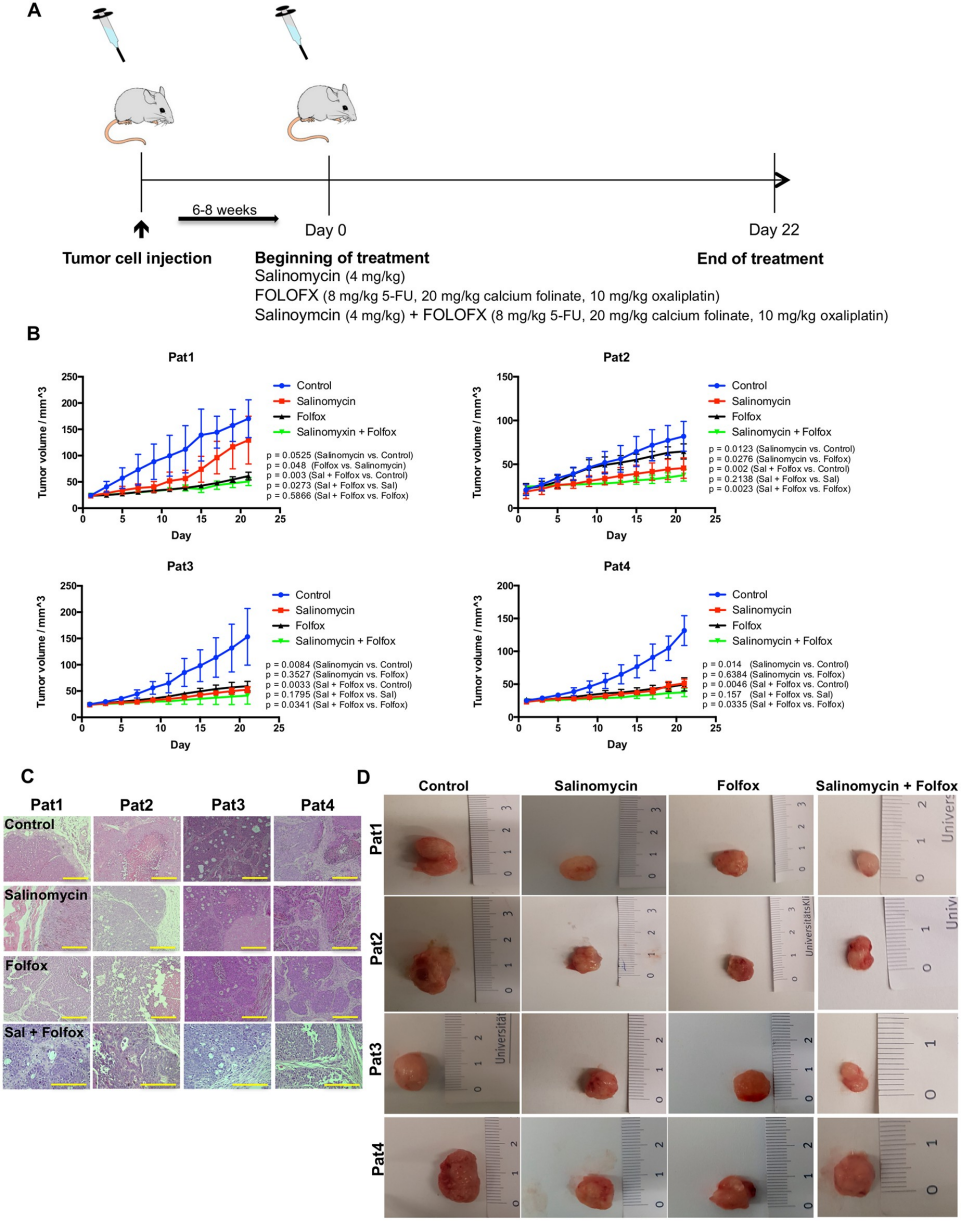
Salinomycin inhibits tumor growth in a patient-derived xenograft model. (A) Subcutaneous colorectal cancer growth in NOD/SCID-IL2RG^null^ mice was induced through injection of TICs into the right flank of the animals. After tumor growth, treatment with either vehicle, salinomycin, or FOLFOX regimen (5-fluorouracil, calcium folinate, and oxaliplatin) was started. (B) After 21 days of treatment, Salinomycin significantly inhibited colorectal cancer growth in the subcutaneous tumor model of patient 2 and was non-inferior in the tumor models of patients 3 and 4. In contrast, FOLFOX therapy was superior compared to salinomycin in the tumor model of patient 1. Combined treatment with salinomycin and FOLFOX resulted in increased anti-tumoral activity in all tumor models. Results are shown as mean tumor volume ± SD (C), H&E stained sections (D), and representative images of explanted tumors of 7 individual experiments. Scale bars = 100 μM.

Next, we applied a patient-derived xenograft model to investigate the effects of salinomycin on colorectal cancer growth *in vivo*. TICs were injected subcutaneously into the flank of NOD/SCID-IL2RG^null^ mice. After successful tumor formation, animals were treated either with vehicle, salinomycin, or FOLFOX (5-FU, folic acid, and oxaliplatin (Fig 4A). Chemotherapy was tolerated by the animals as demonstrated by analysis of body weight during treatment (S6 Fig).

Treatment with salinomycin was superior compared to FOLFOX in one patient-derived xenograft model (patient 1) and equivalent to FOLFOX in two xenograft models (patients 3 and 4). FOLFOX therapy was superior compared to salinomycin in the xenograft model derived from patient 1 (Fig 4B). Combined treatment with salinomycin and FOLFOX resulted in increased anti-tumoral activity compared to FOLFOX alone in all xenograft models. The growth-inhibiting effect of the combination therapy was statistically significant in vivo in TICs derived from patients 2, 3, and 4. Representative images of H&E stained tumor sections and explanted tumors are depicted in Fig 4C+4D.

### Salinomycin inhibits proliferation, induces cell death and abolishes ATP production of human colorectal cancer cells

To delineate the molecular mechanism underlying salinomycin’s potency against human colorectal cancer cells, we assessed the impact of salinomycin on mitochondrial function in three human colorectal cancer cell lines. We hypothesized that salinomycin interferes with mitochondrial function leading to impaired tumor cell survival.

At the outset, we investigated whether treatment with salinomycin inhibits proliferation and survival of three human colorectal cancer cell lines. For this purpose, HT29, SW480, and HCT116 cells were exposed to increasing concentrations of salinomycin (0.1, 0.5, 2, 5, and 10 μM) for 24 hours, and tumor cell proliferation was assessed using the BrdU incorporation assay. As demonstrated in S7A Fig, treatment with salinomycin resulted in a dose-dependent inhibition of tumor cell proliferation.

Next, we analyzed induction of cell death following exposure to salinomycin and measured LDH release in HT29, SW480, and HCT116 cells after 24 hours of treatment. As shown in S7B Fig, salinomycin causes increased LDH release in all three cell lines. We further analyzed induction of apoptosis by measuring the amount of AnnexinV/PI positive cells. Treatment with salinomycin likewise resulted in a dose-dependent induction of apoptosis in all cell lines (S7C Fig).

After confirming the cytotoxic activity of salinomycin in colorectal cancer cells, we investigated the impact of salinomycin on cellular ATP production. As shown in S7D Fig, treatment with increasing concentrations of the compound was associated with decreased cellular ATP levels. Cell viability in these experiments was monitored in parallel (S8 Fig).

### Accumulation of dysfunctional mitochondria and increased production of reactive oxygen species upon salinomycin treatment

**Fig 5:**
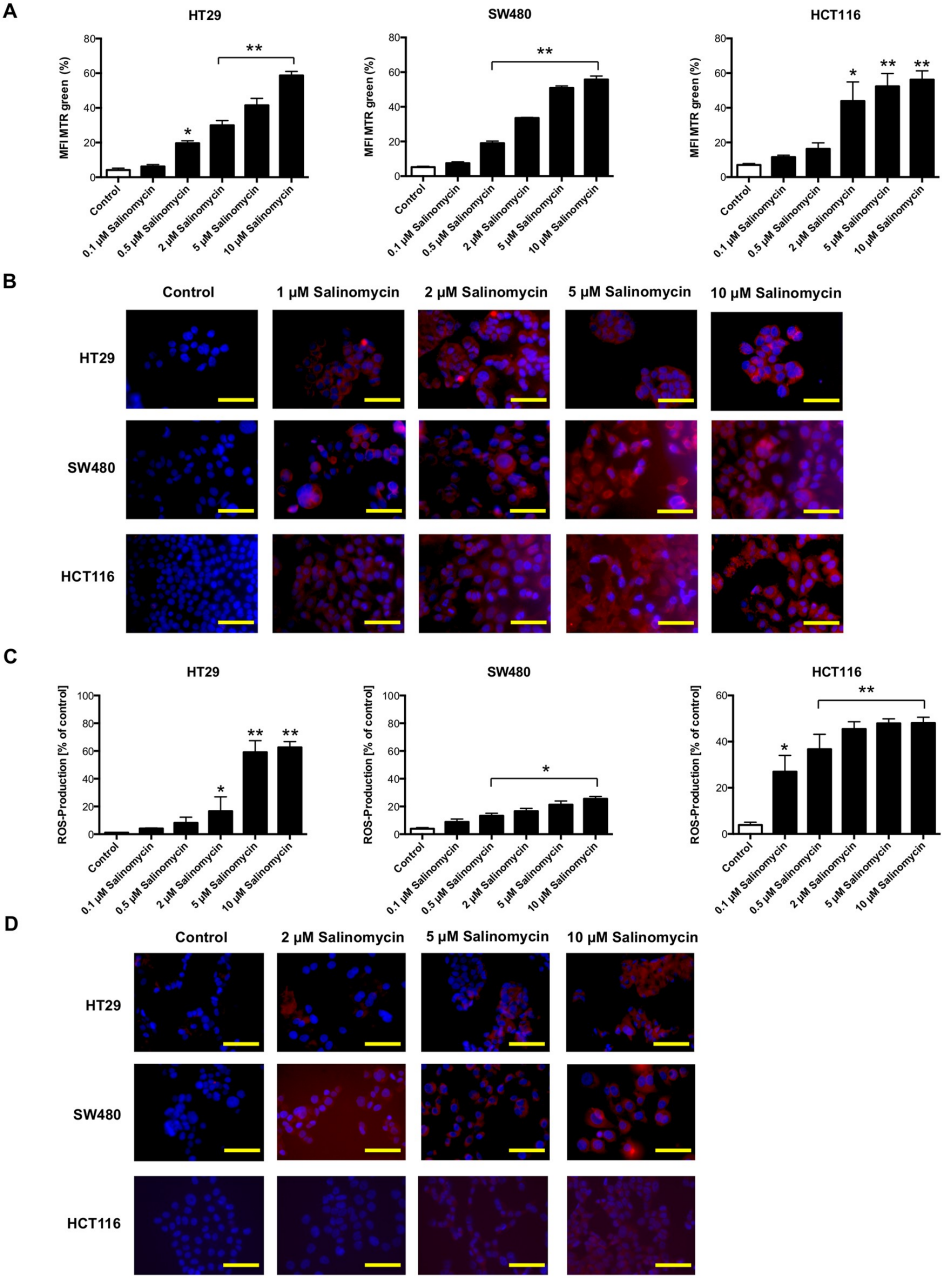
Treatment with salinomycin results in accumulation of dysfunctional mitochondria and increased generation of ROS. (A) Analysis of total mitochondrial mass in HT29, SW480, and HCT116 cells after exposure to increasing concentrations of salinomycin (0.1, 0.5, 2, 5, and 10 μM) was assessed using MTR green. After 24 hours, accumulation of mitochondrial mass was observed in all three cell lines. (B) Accumulation of dysfunctional mitochondria was further visualized applying MRT green immunostaining after 24 hours of treatment. (C) Increased generation of ROS in HT29, SW480, and HCT116 cells after treatment with salinomycin using CM-H2DCFDA staining and analyzed by flow cytometry. (D) Immunostaining of HT29, SW480, and HCT116 cells after exposure of salinomycin confirmed increased ROS generation. Results are displayed as a summary of at least three independent experiments as mean ± SD or as representative image capture by fluorescence microscopy; * *p* < 0.05 compared with control. N = 3 individual experiments. Scale bars = 50 μM.

Based on the observation that reduced ATP levels of tumor cells might be associated with an increased amount of dysfunctional mitochondria (and consequently increased production of reactive oxygen species (ROS) [11]), we analyzed whether treatment with salinomycin results in accumulation of mitochondrial mass. As shown in Fig 5A+5B, treatment with salinomycin for 24 hours results in an increased amount of mitochondrial mass in colorectal cancer cells. Given that accumulation of mitochondrial mass is an indicator of mitochondrial dysfunction, we analyzed the generation of ROS after exposure to salinomycin. Indeed, treatment with salinomycin for 24 hours led to increased ROS generation in HT29, SW480, and HCT116 cells (Fig 5C+5D).

### Exposure to salinomycin inhibits respiratory chain complex II and increases SOD1 expression

Finally, we aimed to investigate whether treatment with salinomycin is related to inhibitory effects on the mitochondrial respiratory chain. Therefore, we analyzed the activity of respiratory chain complexes I, II, and citrate synthase activity. Exposure to salinomycin resulted in inhibition of complex II in all three colorectal cancer cell lines. Function of complex I and citrate synthase activity were unaffected by salinomycin treatment (S9A–S9C Fig).

To correlate these findings with genomic expression patterns of ROS- and cellular repair-related genes, we performed qPCR analysis and analyzed the mRNA expression of superoxide dismutase 1 (SOD1). SOD1 expression is postulated to protect cells from damage caused by ROS [25,26]. As shown in S9D Fig, mRNA expression of SOD1 was strikingly inhibited by salinomycin treatment in a dose-dependent manner.

## Discussion

The basic principles of systemic chemotherapy include classical cytostatic drugs, antibody-, hormone- or immuno-based therapies, and, as of late, targeted therapies. A paradigm shift in basic oncological research relates to the discovery of salinomycin as a specific inhibitor of cancer stem cells [27–30]. This could include treating primary tumor growth, the formation of metastases, and tumor recurrence. The aim of the present pre-clinical study was to investigate the impact of salinomycin on patient-derived colorectal cancer initiating cells. The results confirm that salinomycin exhibits increased anti-cancer stem cell activity compared to 5-FU and oxaliplatin *in vitro*. Furthermore, salinomycin inhibits tumor growth in a patient-derived colorectal cancer xenograft model. Combined treatment with salinomycin, 5-FU, and oxaliplatin resulted in superior activity compared to salinomycin monotherapy.

Salinomycin, initially isolated from *Streptomyces albus* [31], was used over decades in animal farming due to its coccidiostatic activity [32]. Gupta et al. described in 2009 for the first time the inhibitory effects of the compound in breast cancer stem cells [5]. Thereupon, the anti-cancer activity of salinomycin has been studied in detail over the past years [28]. Until now there are no data available analyzing the activity of salinomycin in patient-derived cancer stem cells. For colorectal cancer, the stem-cell specific activity of salinomycin was extrapolated from stem-like cells within immortalized human colorectal cancer cell lines [14–16,33–36]. Therefore, we investigated the activity of salinomycin against three TIC lines derived from patients with colorectal liver metastases and one TIC line derived from a patient with colon cancer *in vitro* and *in vivo*. The obtained findings indicate that salinomycin might display potential for the treatment of colorectal cancer in (pre)clinical practice.

First, we demonstrate that salinomycin reduces viability and inhibits proliferation of all TIC cultures in a dose-dependent manner, whereas 5-FU and partially oxaliplatin stay less active. Furthermore, salinomycin induces apoptosis in TIC cultures in a time-dependent manner. This effect has been observed in colorectal cancer cells and other types of cancer before [10,11,34,37]. Strikingly, salinomycin acts directly inhibitory of cancer stem cells by inhibition of spheroid formation and transcript levels of stem cell-related genes. Particularly, the decreased expression of Lgr5, one of the most important genes for stem cell hierarchy and maintenance in colorectal cancer, underlines the stem cell activity of salinomycin [24,38]. Furthermore, Lgr5 is required for the maintenance of spheroid-derived colorectal cancer cells [39]. The expression of cancer stem cell surface markers, such as CD133, CD44, or EpCam, was not influenced by salinomycin treatment. However, the tumorigenic potential of the TICs used in this study is independent of the expression of stem cell surface markers [18,19]. ALDH1 expression, which has been demonstrated to label colorectal cancer stem cells [23], is also inhibited by salinomycin treatment. Of note, exposure to 5-FU and oxaliplatin did also reduce ALDH1 expression in TICs used in this study.

Second, salinomycin inhibits tumor growth in four patient-derived colorectal cancer xenograft models. Compared to FOLFOX regimen, treatment with salinomycin was superior regarding inhibition of tumor growth in one model and non-inferior in two models. In the xenograft model derived from patient 1, FOLFOX therapy was superior compared to treatment with salinomycin. This inhomogeneous response to salinomycin treatment also reflects the individuality of each tumor. Strikingly, the combination of salinomycin and FOLFOX resulted in increased anti-tumoral activity in all four patient-derived xenograft models. This synergistic effect of salinomycin and FOLFOX might represent the backbone for further pre-clinical investigations. Synergistic effects of salinomycin and other established cytotoxic drugs have been described before [40–42].

The heterogeneous response of salinomycin treatment among the four patient-derived TICs *in vitro* and *in vivo* is explainable by the biological heterogeneity of each tumor and reflects the clinical reality. Similar observations have been made before when the anti-cancer activity of several drugs in patient-derived xenografts were investigated [43–45].

Third, induction of apoptosis by salinomycin is associated with accumulation of dysfunctional mitochondria and ROS, and decreased cellular ATP production in human colorectal cancer cells. It is assumed that cancer cells are dependent on functional mitochondria to maintain their cellular energy production [46]. Indeed, sufficient ATP production as a source for cellular energy is regarded as mandatory for epithelial cancer cells to show migration activity and to form distant metastasis [47]. Furthermore, salinomycin inhibits the activity of complex II, which is a key enzyme in mitochondrial respiration. The accumulation of ROS in colorectal cancer cells might be explained by decreased expression of SOD1, which is assumed to exert protective effects on cells damaged by ROS [25]. In line, inhibition of SOD1 in mice was shown to inhibit angiogenesis and proliferation, making it a potential target of anti-cancer therapies [48].

Of note, we do not imply that inhibition of SOD1 might represent an exclusive mechanism of salinomycin to treat colorectal cancer cells. Inhibition of Wnt-signalling or interference with autophagic flux might also contribute to the mode of action of salinomycin [15,34]. In breast cancer stem cells, salinomycin acts via accumulation in the endoplasmatic reticulum leading to enhanced Ca^2+^ release into the cytosol, which is the initiating event for inhibition of the Wnt signalling pathway [49]. In osteosarcoma and primary breast cancer cells, salinomycin eliminates cancer stem cells by sequestering iron in lysosomes [50]. Thus, the observed effects in this study might be a consequence of the Ca^2+^ increase in the cytoplasm induced by salinomycin accumulation in the endoplasmatic reticulum. Detailed genetic characterization of the patient-derived xenograft model used in this study will provide further insights into the mechanisms of action of salinomycin in colorectal cancer stem cells.

Only rare data exist investigating the pre-clinical usage of salinomycin in men. One case-report describes intravenous administration of salinomycin in a small cohort of patients with metastasized breast, ovarian, and head and neck cancer. After three weeks of treatment, partial tumor and metastases regression has been observed. Interestingly, only minor acute or long-term side effects are described [51]. Despite all justifiable expectations of salinomycin and its clinical applicability to treat colorectal cancer, the potential toxic side effects of the drug cannot be neglected. Accidental severe intoxications in humans and animals have been described [52,53]. Synthesis of structural analogs with an improved activity and reduced toxicity might be an alternative to the original compound [54–56]. We have demonstrated its effectiveness against colorectal cancer cells recently [37]. Thus, semi-synthetic analogs of salinomycin used at lower concentrations show equivalent activity against colorectal cancer cells than salinomycin, which may have potential for reducing the toxic side effects of salinomycin.

## Conclusions

In summary, the results of this study demonstrate the activity of salinomycin in a patient-derived pre-clinical model for colorectal cancer *in vitro* and *in vivo*. Thus, salinomycin remains a candidate for the (pre)clinical usage to treat colorectal cancer. Combined treatment with salinomycin and FOLFOX might be the best applicability. Further studies are needed to discover the best drug dose-intensity and potential toxic effects in normal, non-cancerous cells.

## Methods

### Cell lines and culture

We used four TIC spheroid cultures obtained from patients with metastasized colorectal cancer in accordance with the Declaration of Helsinki and after approval of the Ethics Commission of the University Hospital of Heidelberg. Informed consent was obtained from all patients. Patient characteristics are summarized in S1 Table. Establishment and growth of spheroid cultures was described extensively before [18,19]. In brief, tumor tissue was minced and enzymatically digested with dispase and cultured in ultra-low attachment flasks (Corning). This results in the death of non-cancerous cells and formation of 3D spheroid cells. Cell culture was continued under serum-free conditions in advanced D-MEM/F-12 medium supplemented with glucose to 0.6%, 1% penicillin/streptomycin, 1 mM L-glutamine (Invitrogen), 4 μg/ml heparin, 5 mM HEPES, 4 mg/ml BSA (Sigma-Aldrich), 10 ng/ml fibroblast growth factor (FGF) basic, and 20 ng/ml epidermal growth factor (EGF; R&D Systems). Growth factors were added every 4 days and medium was changed every 7 to 14 days [18]. The cells were incubated at 37 °C and 5% CO_2_. Authentication of the spheroid cultures and exclusion of contamination was performed by Multiplexion (http://www.multiplexion.de/en/). For immunophenotyping, the cells were dissociated with accutase and stained with anti-human CD133 (clone AC133, Miltenyi), anti-human CD44 (clone G44-26, Becton Dickinson), and anti-human CD326 (EpCam, clone 9C4, BioLegend). Surface marker expression was analyzed by flow-cytometry (FACSCalibur, Becton Dickinson). In this study, the same spheroid cultures were used as described and characterized in detail before [18,19]. In all experiments, TICs were used and plated as spheres. Therefore, TICs were dissociated with accutase, stained with Trypan Blue solution and counted using Neubauer chambers. In all experiments with TICs, non-adherent cell culture material was used.

The three human CRC cell lines HT29, SW480, and HCT116 were used to investigate the molecular mechanism of salinomycin *in vitro*. Cells were obtained from American Type Culture Collection (ATCC numbers: HTB38, CCL-228, and CCL-247). SW480 and HT29 cells were cultured in RPMI 1640 medium (Invitrogen); HCT116 cells were cultured in DMEM High Glucose medium (Sigma Aldrich). All media were supplemented with 10% fetal calf serum, penicillin (50 U/ml) and streptomycin (50 mg/l). The cells were incubated at 37 °C and 10% CO_2_.

### Chemicals and antibodies

Salinomycin sodium salt was purchased from Sigma Aldrich and dissolved in dimethyl sulfoxide (DMSO) to obtain a stock solution (10 mM). The DMSO concentration in the medium was 0.05% when using the compound at a 0.5 μM concentration. For the experiments, Salinomycin-Na (sodium salt) has been used, abbreviated as “salinomycin”.

5-fluorouracil (5-FU), calcium folinate, and oxaliplatin were obtained from the pharmacy of the University Hospital of Heidelberg. All compounds were diluted with phosphate-buffered saline (PBS) to receive appropriate working solutions [10,57]. Stock solutions were stored at -20 °C.

### Cell proliferation and viability assay

TICs or the three human colorectal cancer cell lines HT29, SW480, and HCT116 (5 x 10^3^ each) were cultured in 96-well flat bottom plates. Cells were exposed to increasing concentrations of salinomycin, 5-FU, or oxaliplatin (0.1 μM, 0.5 μM, 1 μM, 2 μM, 5 μM, and 10 μM) for 24–48 hours. The effect of the evaluated compounds on the cell number was assessed using the WST-1 assay (Sigma Aldrich) according to the manufaturer’s instructions. Cell proliferation was assessed using the bromodeoxyuridine (BrdU) ELISA kit (Sigma Aldrich), according to the manufacturer’s instructions. Additionally, cell numbers were analyzed applying the CellTiter-Glo Viability Assay (Promega). This assay is based on measuring a luminescence signal proportional to the ATP content present in healthy cells. The luminescence signal was quantified by a microplate luminometer as recommended by the manufacturer’s instructions.

### Cell death

Induction of TICs’ death after exposure to salinomycin, 5-FU, or oxalipaltin was analyzed measuring DNA fragmentation. 0.5x10^6^ cells were seeded in 6-well plates and exposed to increasing concentrations of salinomycin, 5-FU, or oxaliplatin (1 μM, 2 μM, 5 μM, and 10 μM) for 24 and 48 hours. After permeabilisation, the cells were stained with the DNA intercalating dye propidium iodide (PI). The DNA profile was measured by flow-cytometry and the apoptotic cells were defined as the sub G1 fraction as previously described [21]. During analysis, gates were set to exclude small particles with low DNA content. To receive a better resolution between the two peaks in cells growing in suspension, analysis was performed with a logarithmic amplification of DNA fluorescence [21]. Induction of apoptosis of TICs was further assessed by AnnexinV analysis (BD Biosciences). TICs were treated either with increasing concentrations of salinomycin, 5-FU, or oxaliplatin (1 μM, 2 μM, 5 μM, and 10 μM) for 24 hours. Cells were dissociated with accutase and stained according to the manufacturers’ instructions. Induction of apoptosis was measured by flow-cytometry.

Alternatively, cell death of HT29, SW480, and HCT116 cells was further analyzed applying the Lactate dehydrogenase (LDH) Cytotoxicity Assay Kit (Thermo Fisher) following the manufacturer’s instructions as previously described [37].

### Spheroid formation assay

Spheroid formation assay was conducted to assess the long-lasting effects of salinomycin compared to FOLFOX treatment on the four TIC cultures used in this study. Therefore, TIC spheres were dissociated with accutase and 2x10^4^ cells were seeded in a 24-well plate. Cells were treated with increasing concentrations of salinomycin, 5-FU, or oxaliplatin (1 μM, 2 μM, 5 μM, and 10 μM) and observed daily. Medium was not changed, but growth factors were added every three days. Spheroid formation was assessed after 21 days using phase-contrast microscopy.

### Cancer stem cell activity assay

The anti-cancer stem cell activity of salinomycin was analyzed applying the ADELFOUR kit (Stem Cell Technologies) according to the manufacturer’s protocol. After exposure to salinomycin, 2x10^5^ TICs were either incubated with the ALDH1 inhibitor diethylaminobenzaldehyde (DEAB) and the ALDH1 substrate BODIPY-amino acetaldehyde or only BODIPY-amino acetaldehyde. DEAB-treated cells served as a control to set the ALDH1^+^ region for each sample using a Becton Dickinson flow cytometer as described before [58].

### RNA isolation and real-time PCR

Total RNA from tumor cells was isolated by an RNA extraction kit (Qiagen) and cDNA synthesis and real-time (RT)-PCR were performed using the first strand cDNA synthesis kit (Fermentas) and SYBR Green Master Mix kit (Roche) applying specific primers (Life Technologies) for human leucine-rich-repeat-containing G-protein-coupled-receptor 5 (Lgr5) and superoxide dismutase 1 (SOD1). Transcript levels of the gene of interest were normalized to the expression of glyceraldehy-3-phosphat-dehydrogenase (GAPDH). Primer sequences are listed in S2 Table.

### Analysis of ATP production

Cellular ATP was quantified using a luciferase-based assay (Promega) according to the manufacturer’s protocol. Cell viability in these experiments was monitored in parallel using the WST-1 assay as described above.

### Mitochondrial assays and measurement of ROS generation

Mitochondrial mass was analyzed by flow cytometry or fluorescence microscopy using MitoTracker Green FM (MTR green) staining (Molecular Probes, Eugene, OR, USA), according to the manufacturer’s instructions and described in [10]. Complex I and complex II activity was measured using the Complex I and II Enzyme Activity Microplate Assay Kits (Abcam) according to the manufacturer’s protocols and described in [59]. Citrate synthase activity was measured using the Citrate Synthase Assay Kit (Sigma).

The ROS generation was determined by flow cytometry, applying 5-(and-6)-chloromethyl-2′,7′-dichlorodihydrofluorescein diacetate, acetyl ester (CM-H2DCFDA) staining (Invitrogen) according to the manufacturer’s instructions and described in [10]. The ROS generation was further analyzed by applying the MitoTracker Red CM- H_2_-Xros Kit (Invitrogen) according to the manufacturer’s instructions.

### Animal model and treatment

Animal experiments were carried out in 6–10 week-old NOD/SCID-IL2RG^null^ mice purchased from Charles River Laboratories. Animals were housed under specific-pathogen-free conditions in groups of four with free access to food and water under constant environmental conditions with a 12-hour day-night-cycle. Isoflurane was used for inhalation anesthesia. At the start of the experiments, animals weighed 26.7 g ± 1.4 (mean ±SD). For the patient-derived xenograft model, 1 x 10^6^ TICs were injected in 50 μl Matrigel (BD Biosciences) into the right flank under inhalation anaesthesia. Following successful tumor formation after 6–8 weeks, animals were randomized into four treatment groups containing seven mice each. The animals were treated daily at 09:00 a.m. in the home cage either with corn oil (control group), 4 mg/kg salinomycin, FOLFOX (8 mg/kg 5-FU + 20 mg/kg calcium folinate (on six consecutive days) and 10 mg/kg oxaliplatin (once a week)) intraperitoneally, or a combination of salinomycin and FOLFOX [60]. Injection of chemotherapy was performed without anaesthesia. Animal health was monitored twice a day daily. Animals were weighed every second day. Humane endpoints were defined as tumor size > than 15 mm in diameter, tumor ulceration, reduced activity of the animal, weight loss > than 20% of the original weight, reduced softness of the coat, and abnormal behavior. No adverse events were observed. Tumor volume was assessed daily during chemotherapy for a total of 21 days. The maximum tumor size achieved during the study was 13.9 x 14.9 mm. Euthanasia was performed by cervical dislocation. The local animal care committee (Regierungspräsidium Karlsruhe) approved all experiments. The experiments were carried out in accordance with the criteria outlined in the “Guide for the Care and Use of Laboratory Animals” by the National Academy of Sciences. All efforts were made to minimize suffering.

### Immunohistochemistry

Fixed, paraffin-embedded tissue samples were cut into sections of 5 μm and routine hematoxylin and eosin (H&E) staining was performed to evaluate histomorphological features.

### Statistical analysis

Statistical analysis was performed using GraphPadPrism 6. Student’s t-test or ANOVA analysis were applied as appropriate.

Differences were regarded statistically significant with p<0.05 compared to untreated (indicated as “control”) or treated cells. Results were expressed as mean ± standard error of the mean (SEM) of at least three independent experiments.

All data are within the paper and its Supplementary Data files.

## Supporting information

S1 Fig Salinomycin reduces viability of colorectal cancer TICs. TIC cultures from patients1-4 were cultured in the absence or presence of increasing concentrations of salinomycin, 5-fluorouracil, and oxaliplatin (1, 2, 5, and 10 μM) for 24 and 48 hours. Tumor cell viability was assessed applying the CellTiter-Glo Viability Assay. Results are shown as summary of n = 4 independent experiments as mean ± SEM. * p < 0.05, ** p < 0.001 compared with salinomycin treatment.
(TIF)

S2 Fig Induction of apoptosis in TICs after exposure to salinomycin, 5-FU, and oxaliplatin. TIC derived from patients1-4 were cultured in the absence or presence of increasing concentrations of salinomycin, 5-fluorouracil, and oxaliplatin (1, 2, 5, and 10 μM) for 24 hours. Induction if apoptosis was analyzed using SubG1 or AnnexinV-FITC and PI staining and cells were analyzed by flowcytometry. Results are shown as linear amplification of DNA fluorescence (A) or as summary of n = 3 independent experiments as mean ± SEM (B). * p < 0.05, ** p < 0.001 compared with salinomycin treatment.
(TIF)

S3 Fig Preserved spheroid formation of TICs after exposure to 5-fluorouracil. TIC cultures from patients1-4 were cultured in the absence or presence of increasing concentrations of 5-fluorouracil (5-FU; 1, 2, 5, and 10 μM) for 21 days. Cell morphology and sphere formation capacity was assessed daily and cell cultures were documented after end of treatment. Results are shown as representative images (n = 3 individual experiments) of treated TIC with salinomycin. Scale bars = 100 μM.
(TIFF)

S4 Fig Preserved spheroid formation of TICs after exposure to oxaliplatin. TIC cultures from patients1-4 were cultured in the absence or presence of increasing concentrations of oxaliplatin (Oxa; 1, 2, 5, and 10 μM) for 21 days. Cell morphology and sphere formation capacity was assessed daily and cell cultures were documented after end of treatment. Results are shown as representative images (n = 3 individual experiments) of treated TIC with salinomycin. Scale bars = 100 μM.
(TIFF)

S5 Fig Impact of Salinomycin on stem cell marker surface expression of colorectal cancer-derived TICs. Colorectal cancer-derived TICs were exposed to salinomycin (1, 2, 5, and 10 μM) for 24 hours. Expression of the stem cell surface markers CD133, CD44, and EpCam were analyzed by flow-cytometry. Results are shown as representative images (n = 3 individual experiments) of treated TIC with salinomycin.
(TIFF)

S6 Fig Body weight of the animals after treatment. Effect of Salinomycin treatment on body weight (g) of mice in each group.
(TIFF)

S7 Fig Salinomycin inhibits proliferation, induces cell death and reduces ATP levels in human colorectal cancer cell lines. HT29, SW480, and HCT116 cells were cultured in in the absence or presence of increasing concentrations of salinomycin (0.1, 0.5, 2, 5, and 10 μM) for 24 hours. Tumor cell proliferation was assessed using the BrdU incorporation assay (A). Cell death was determined by LDH release assay (B). Induction if apoptosis was analyzed using AnnexinV-FITC and PI staining and cells analyzed by flowcytometry (C). Intracellular ATP levels were assessed applying a luciferase-based ATP assay (D). Results are displayed as a summary of n = 3 independent experiments as mean ± SD; * *p* < 0.05 compared with control.
(TIFF)

S8 Fig Monitoring of cell viability during analysis of cellular ATP levels. Cell viability during analysis of cellular ATP levels was monitored using the WST-1 assay in parallel. Results are displayed as a summary of n = 3 independent experiments as mean ± SD; * *p* < 0.05, ** p < 0.001 compared with control.
(TIFF)

S9 Fig Salinomycin inhibits activity of complex II and reduces the mRNA expression of SOD1. Analysis of complex I (A), II (B), and citrate synthase activity (C) after exposure of HT29, SW480, and HCT116 cells after treatment with 2 and 10 μM salinomycin for 24 hours. mRNA expression of SOD1 in HT29, SW480, and HCT116 cells after exposure to increasing concentrations of salinomycin (0.1, 0.5, 2, 5, and 10 μM) for 24 hours was measured by qRT-PCR. Results are displayed as a summary of n = 3 independent experiments as mean ± SD; * *p* < 0.05, ** p < 0.001 compared with control.
(TIFF)

S1 Table Patient characteristics. (TIFF)

S2 Table Primer sequences of human GAPD, Lgr5, and SOD1. (TIFF)

## Abbreviations

Lgr5: leucine-rich-repeat-containing G-protein-coupled-receptor 5
ROS: reactive oxygen species
SOD1: superoxide dismutase 1
