# Supplementary figures and images for: Salinomycin: Anti-tumor activity in a pre-clinical colorectal cancer model
Source: PLoS One. 2019 Feb 14;14(2):e0211916. doi: 10.1371/journal.pone.0211916 (PMC6375586; doi:10.1371/journal.pone.0211916)

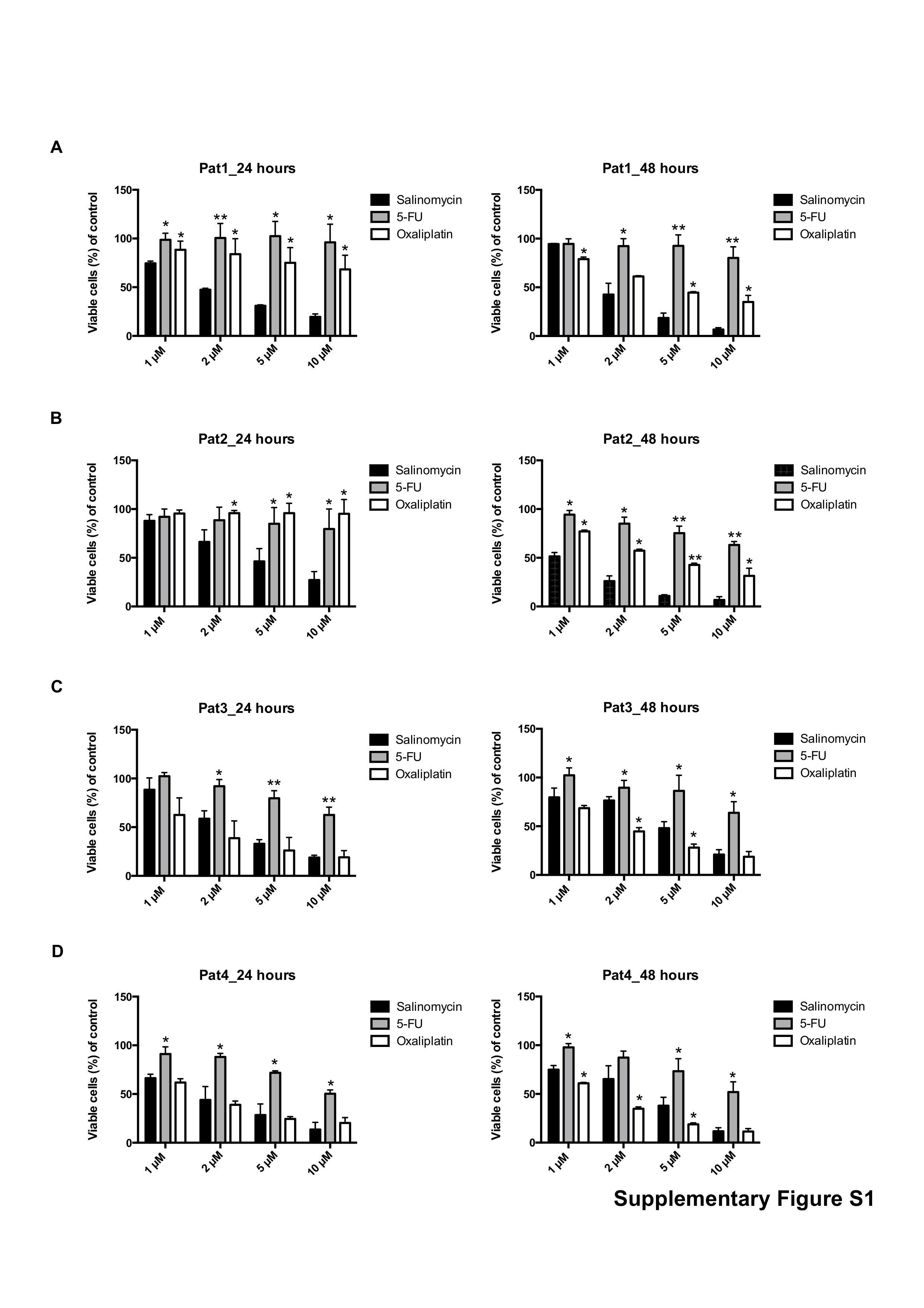

Supplement: S1 Fig — TIC cultures from patients1-4 were cultured in the absence or presence of increasing concentrations of salinomycin, 5-fluorouracil, and oxaliplatin (1, 2, 5, and 10 μM) for 24 and 48 hours. Tumor cell viability was assessed applying the CellTiter-Glo Viability Assay. Results are shown as summary of n = 4 independent experiments as mean ± SEM. * p < 0.05, ** p < 0.001 compared with salinomycin treatment. (TIF) [file pone.0211916.s001.tif]

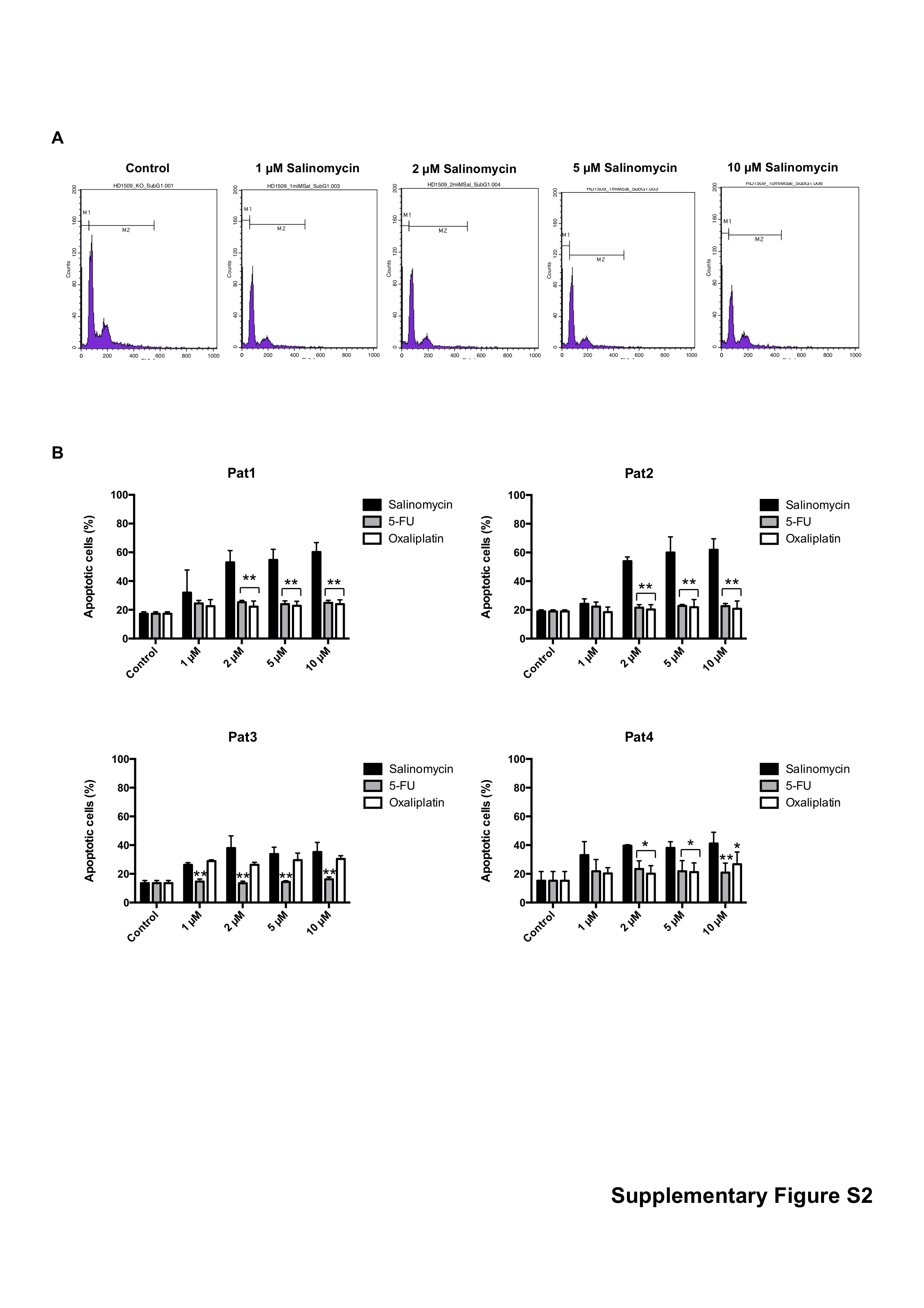

Supplement: S2 Fig — TIC derived from patients1-4 were cultured in the absence or presence of increasing concentrations of salinomycin, 5-fluorouracil, and oxaliplatin (1, 2, 5, and 10 μM) for 24 hours. Induction if apoptosis was analyzed using SubG1 or AnnexinV-FITC and PI staining and cells were analyzed by flowcytometry. Results are shown as linear amplification of DNA fluorescence (A) or as summary of n = 3 independent experiments as mean ± SEM (B). * p < 0.05, ** p < 0.001 compared with salinomycin treatment. (TIF) [file pone.0211916.s002.tif]

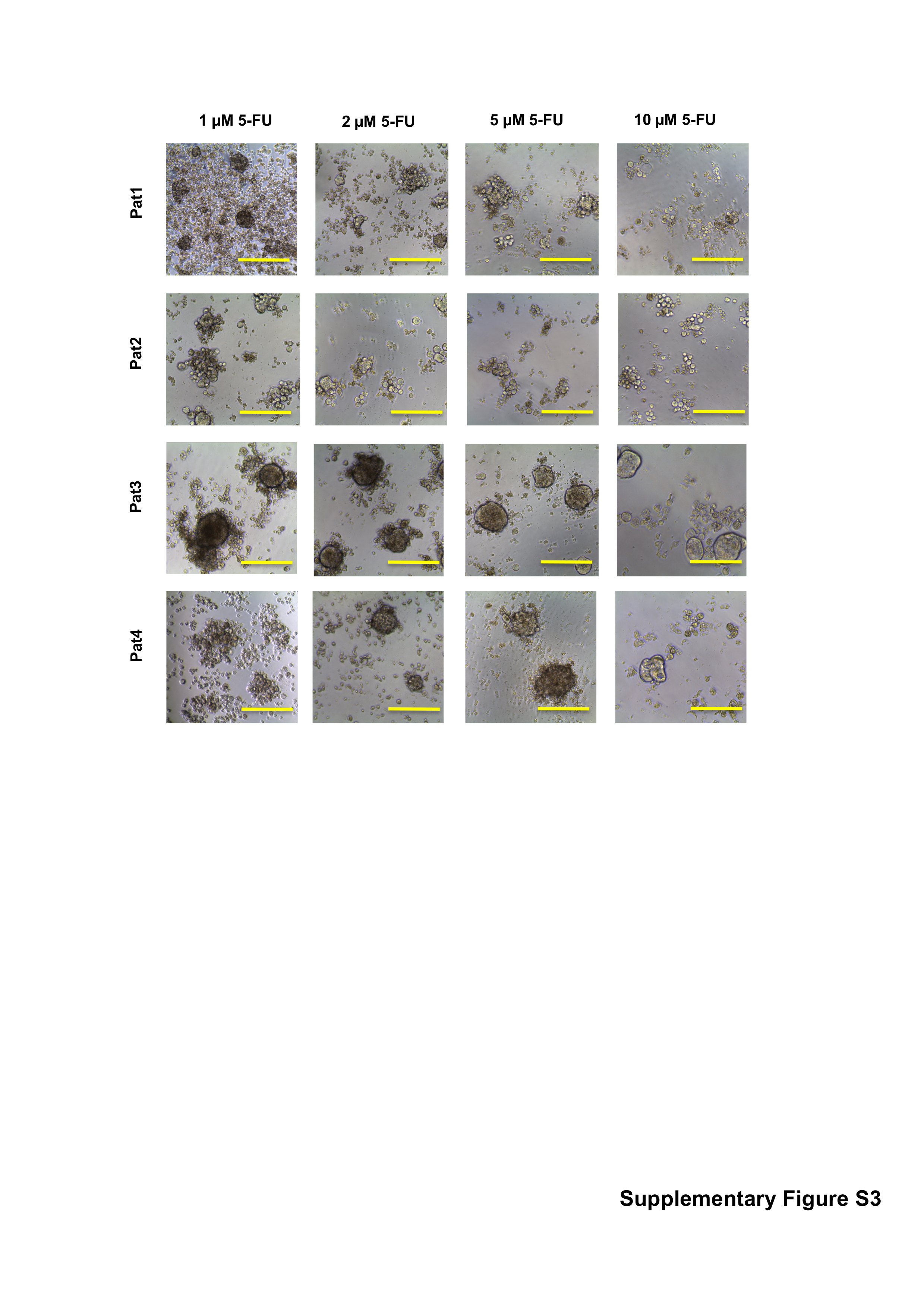

Supplement: S3 Fig — TIC cultures from patients1-4 were cultured in the absence or presence of increasing concentrations of 5-fluorouracil (5-FU; 1, 2, 5, and 10 μM) for 21 days. Cell morphology and sphere formation capacity was assessed daily and cell cultures were documented after end of treatment. Results are shown as representative images (n = 3 individual experiments) of treated TIC with salinomycin. Scale bars = 100 μM. (TIFF) [file pone.0211916.s003.tiff]

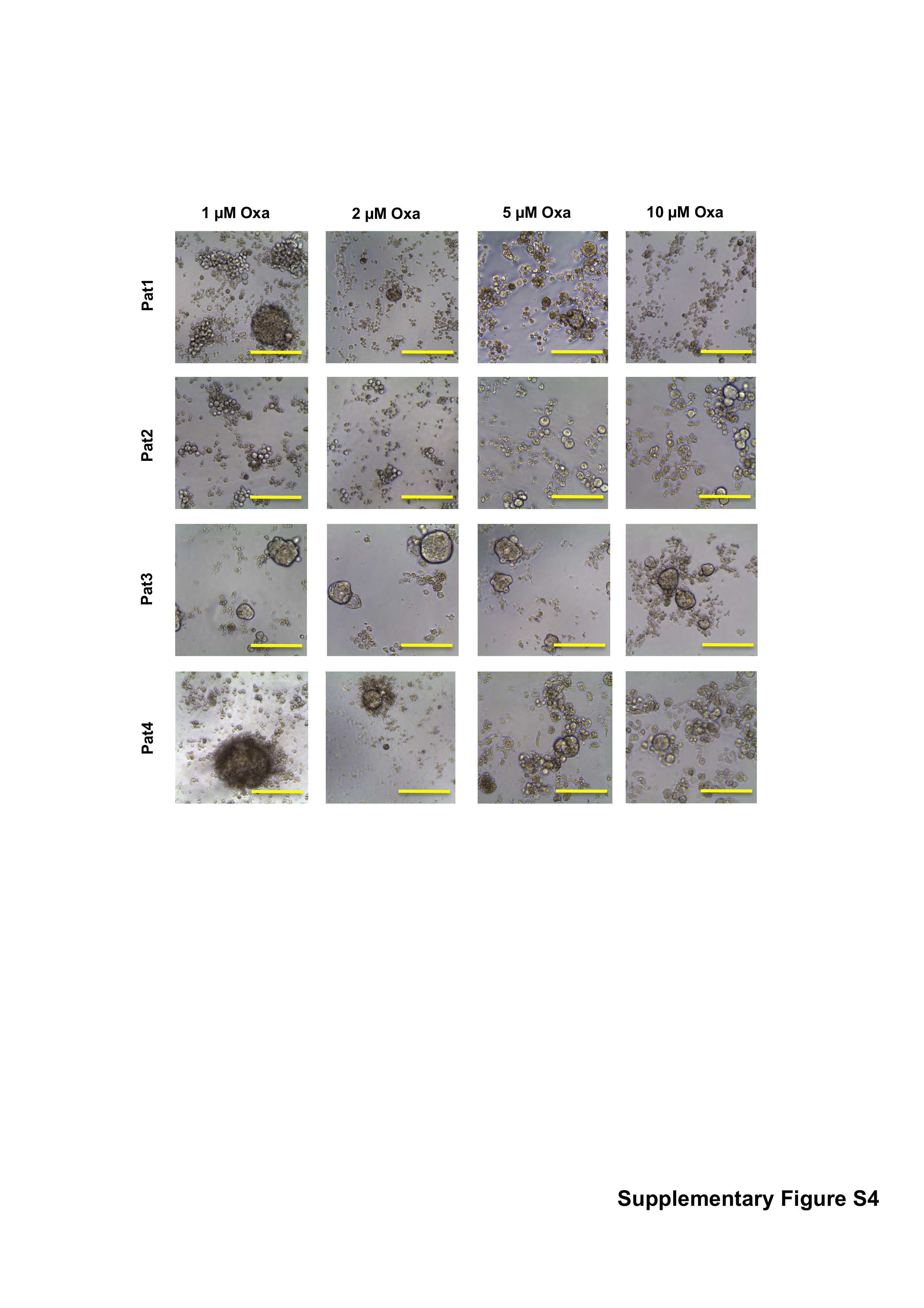

Supplement: S4 Fig — TIC cultures from patients1-4 were cultured in the absence or presence of increasing concentrations of oxaliplatin (Oxa; 1, 2, 5, and 10 μM) for 21 days. Cell morphology and sphere formation capacity was assessed daily and cell cultures were documented after end of treatment. Results are shown as representative images (n = 3 individual experiments) of treated TIC with salinomycin. Scale bars = 100 μM. (TIFF) [file pone.0211916.s004.tiff]

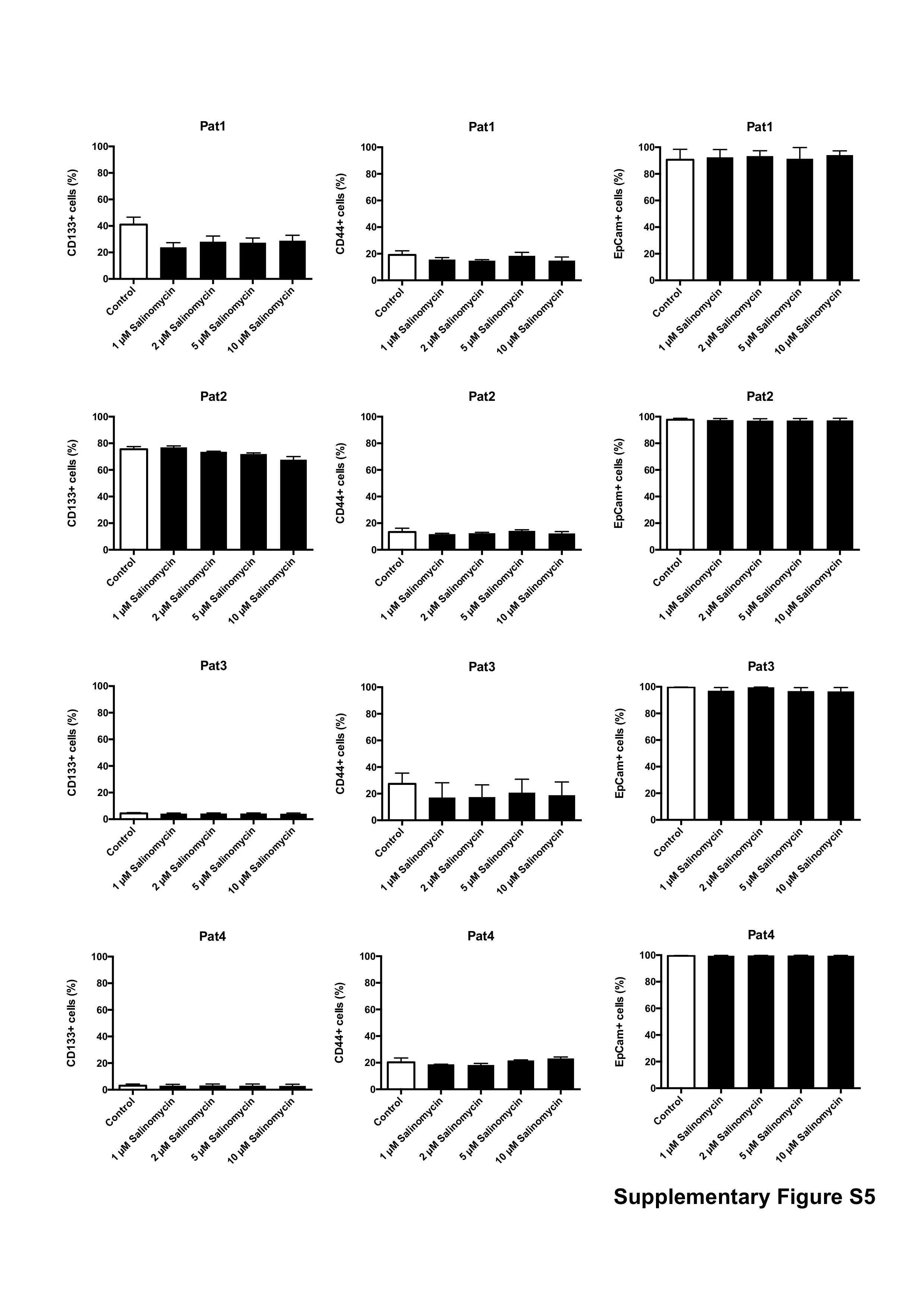

Supplement: S5 Fig — Colorectal cancer-derived TICs were exposed to salinomycin (1, 2, 5, and 10 μM) for 24 hours. Expression of the stem cell surface markers CD133, CD44, and EpCam were analyzed by flow-cytometry. Results are shown as representative images (n = 3 individual experiments) of treated TIC with salinomycin. (TIFF) [file pone.0211916.s005.tiff]

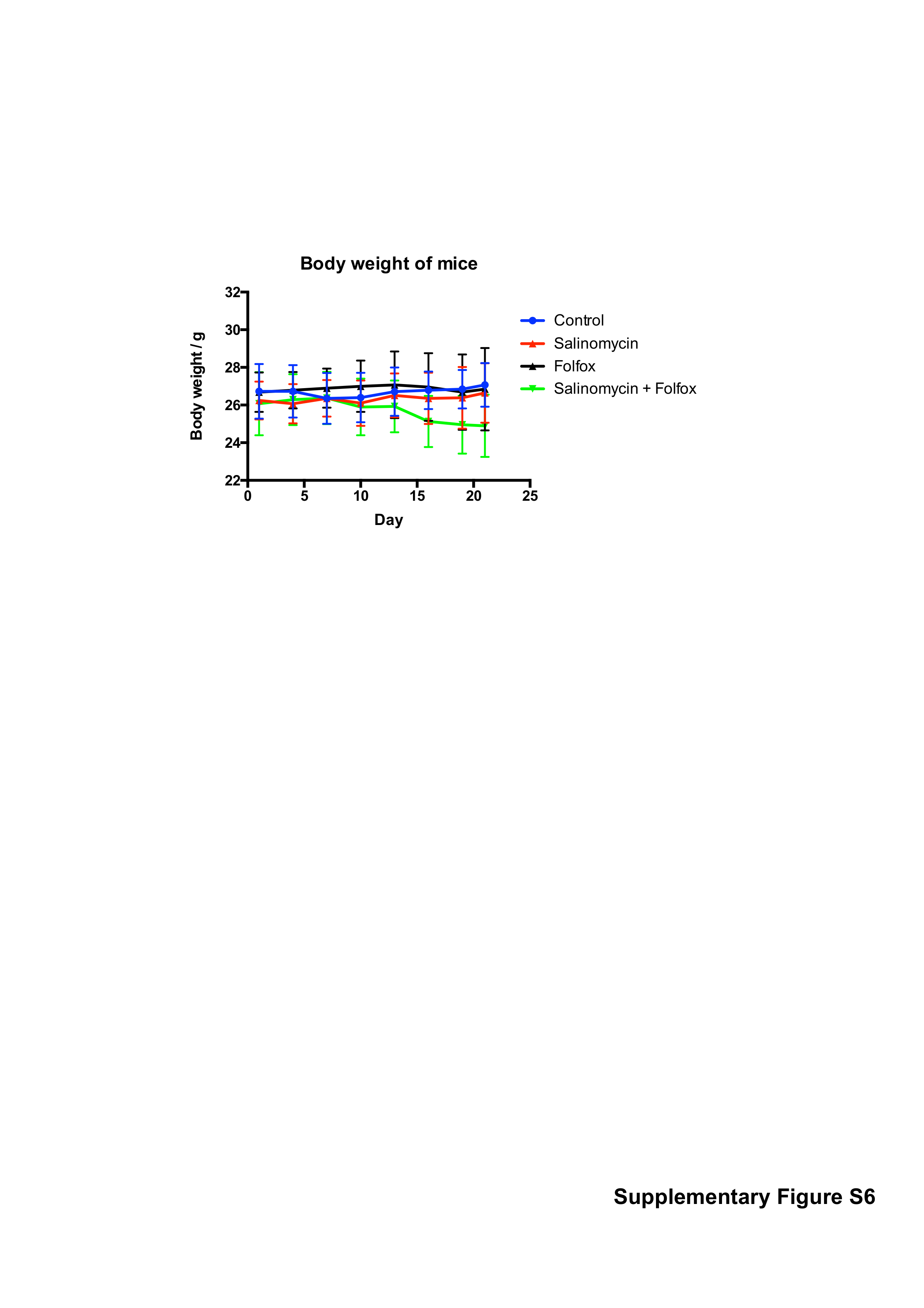

Supplement: S6 Fig — Effect of Salinomycin treatment on body weight (g) of mice in each group. (TIFF) [file pone.0211916.s006.tiff]

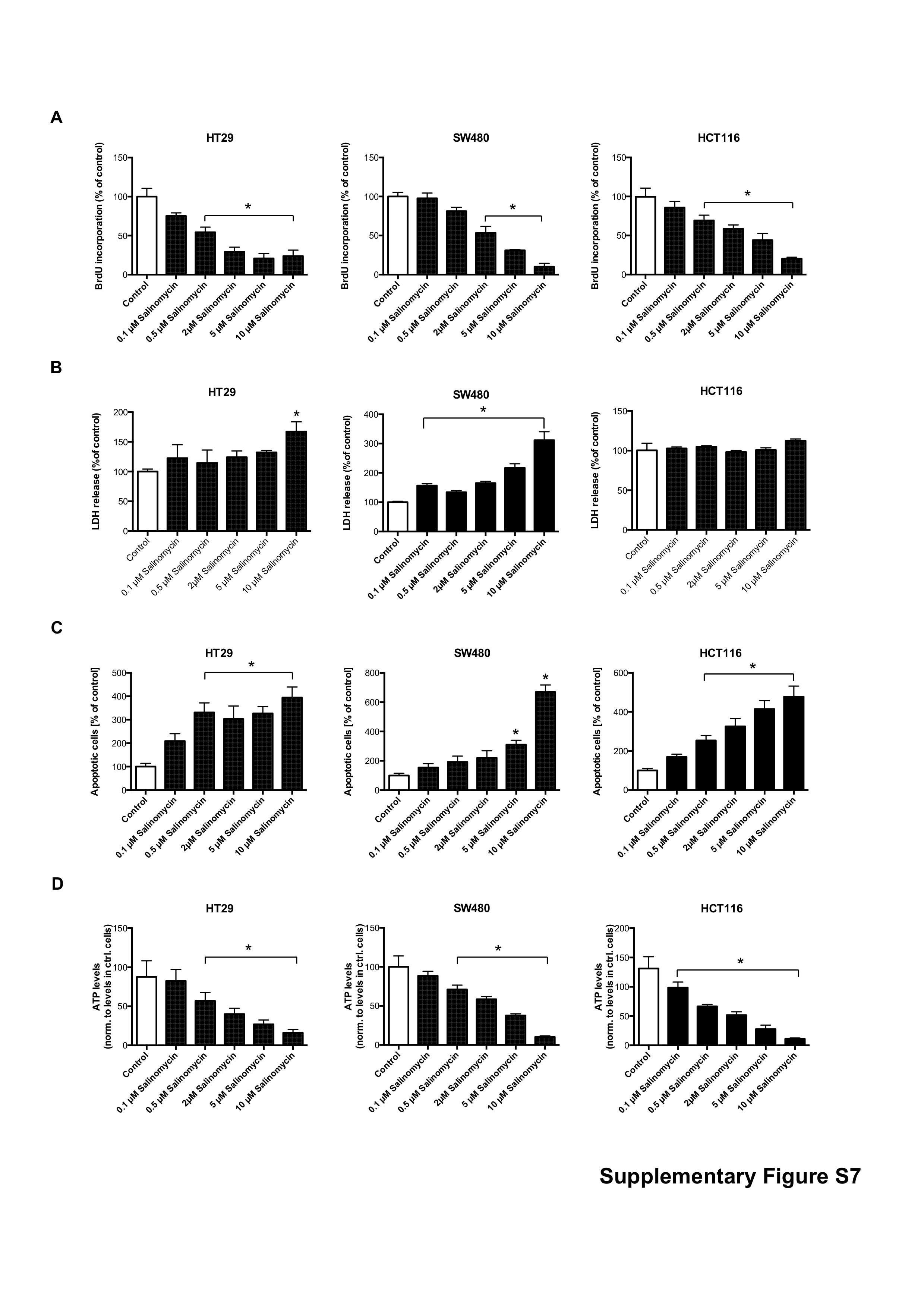

Supplement: S7 Fig — HT29, SW480, and HCT116 cells were cultured in in the absence or presence of increasing concentrations of salinomycin (0.1, 0.5, 2, 5, and 10 μM) for 24 hours. Tumor cell proliferation was assessed using the BrdU incorporation assay (A). Cell death was determined by LDH release assay (B). Induction if apoptosis was analyzed using AnnexinV-FITC and PI staining and cells analyzed by flowcytometry (C). Intracellular ATP levels were assessed applying a luciferase-based ATP assay (D). Results are displayed as a summary of n = 3 independent experiments as mean ± SD; * p < 0.05 compared with control. (TIFF) [file pone.0211916.s007.tiff]

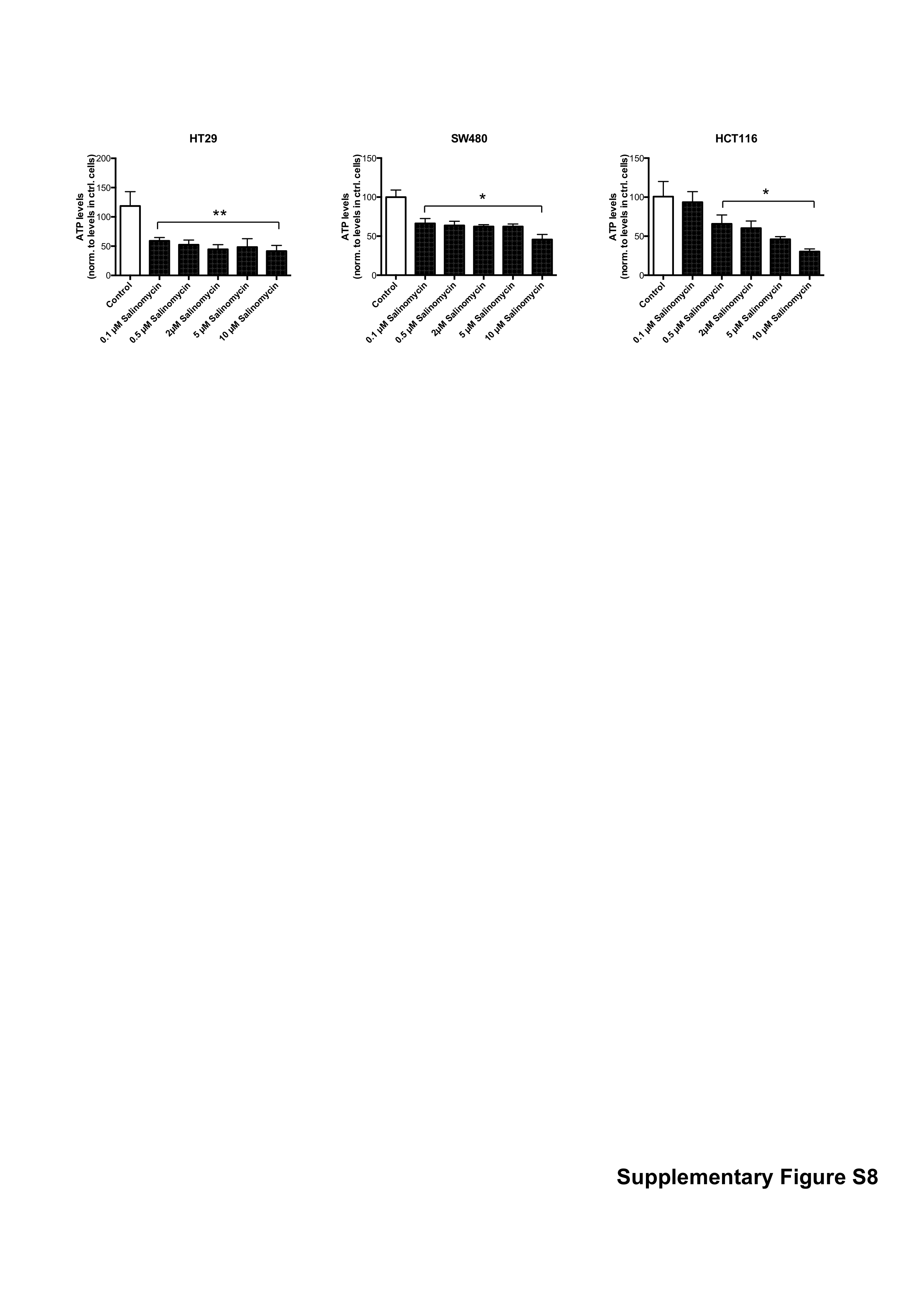

Supplement: S8 Fig — Cell viability during analysis of cellular ATP levels was monitored using the WST-1 assay in parallel. Results are displayed as a summary of n = 3 independent experiments as mean ± SD; * p < 0.05, ** p < 0.001 compared with control. (TIFF) [file pone.0211916.s008.tiff]

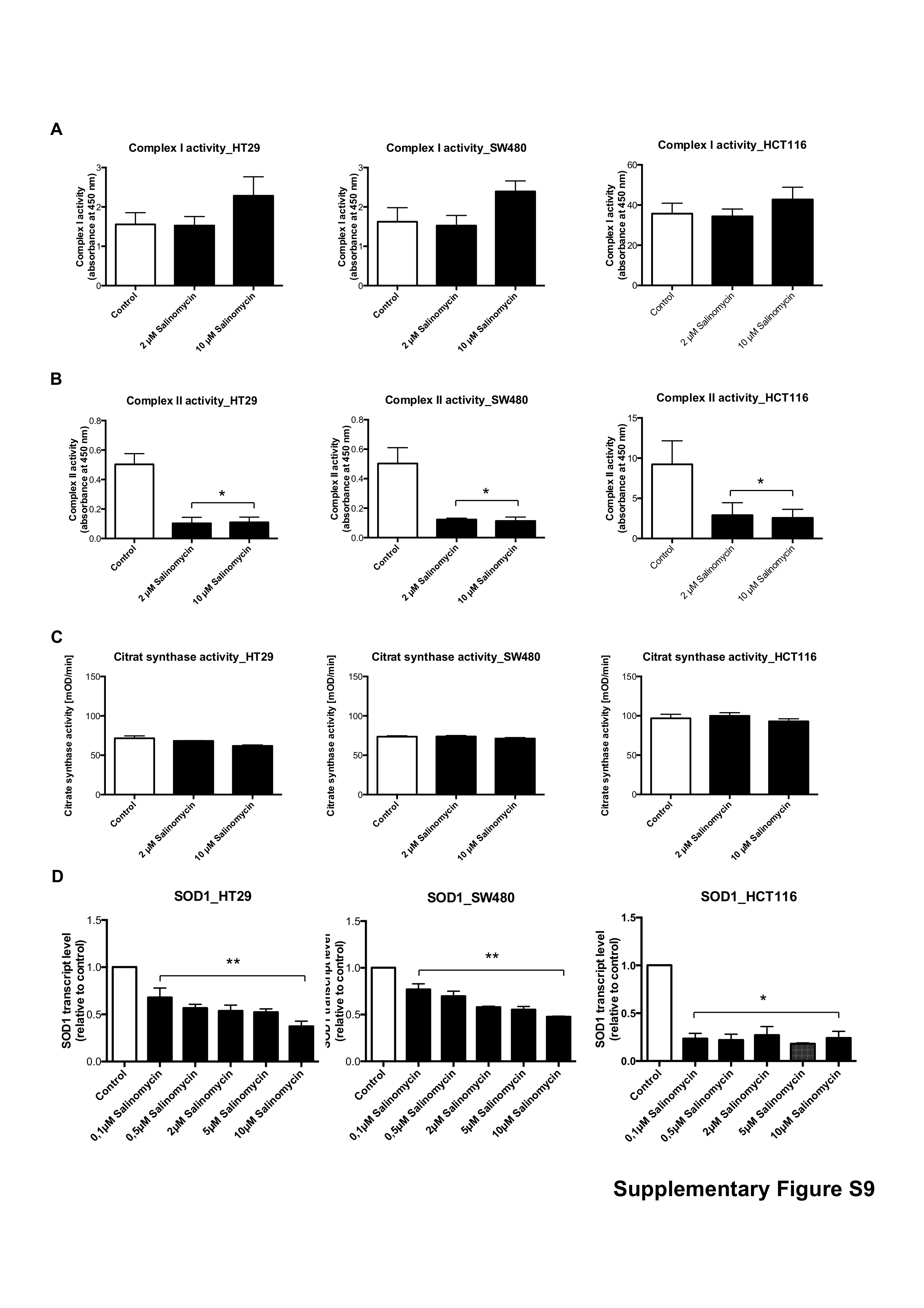

Supplement: S9 Fig — Analysis of complex I (A), II (B), and citrate synthase activity (C) after exposure of HT29, SW480, and HCT116 cells after treatment with 2 and 10 μM salinomycin for 24 hours. mRNA expression of SOD1 in HT29, SW480, and HCT116 cells after exposure to increasing concentrations of salinomycin (0.1, 0.5, 2, 5, and 10 μM) for 24 hours was measured by qRT-PCR. Results are displayed as a summary of n = 3 independent experiments as mean ± SD; * p < 0.05, ** p < 0.001 compared with control. (TIFF) [file pone.0211916.s009.tiff]

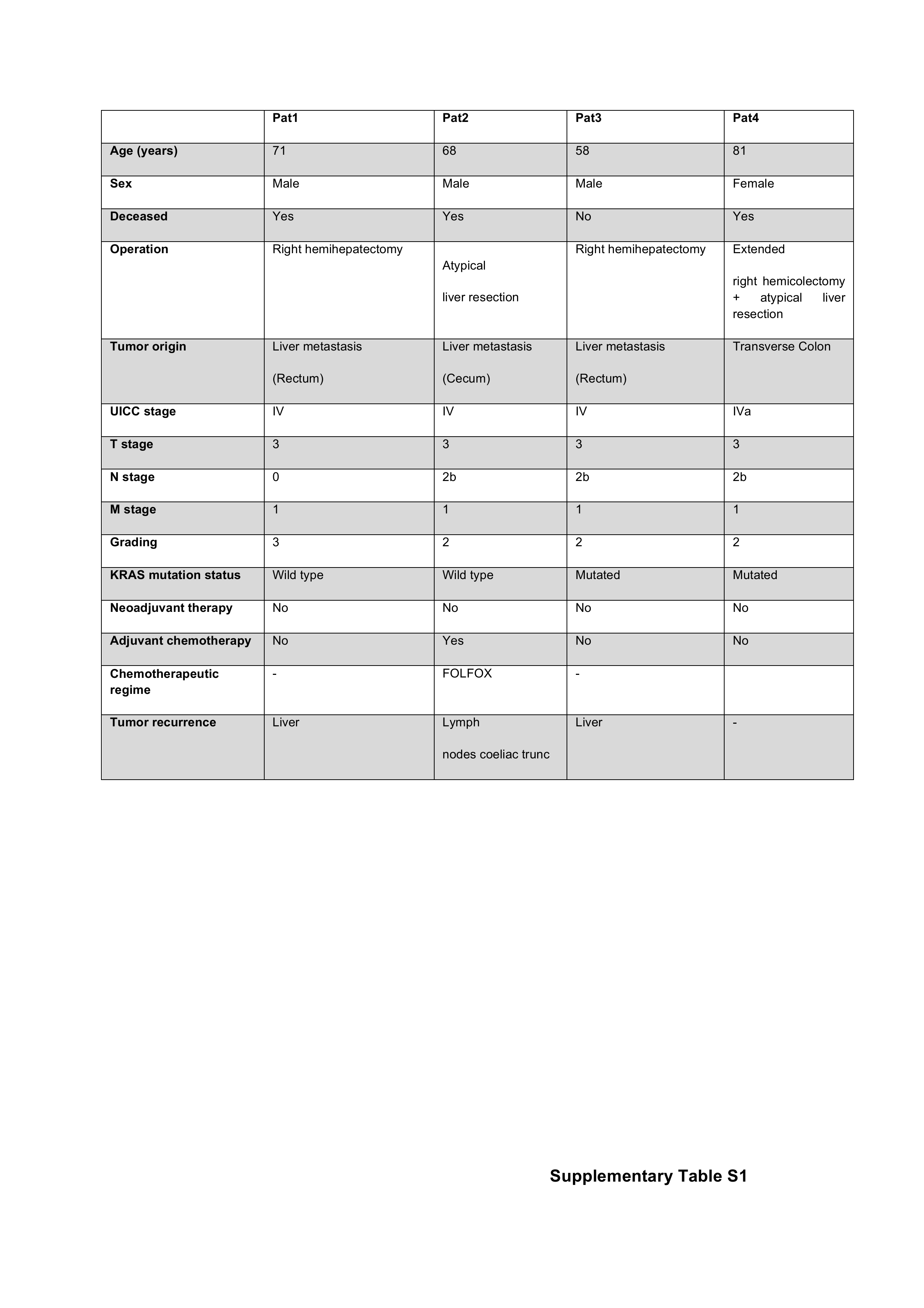

Supplement: S1 Table — (TIFF) [file pone.0211916.s010.tiff]

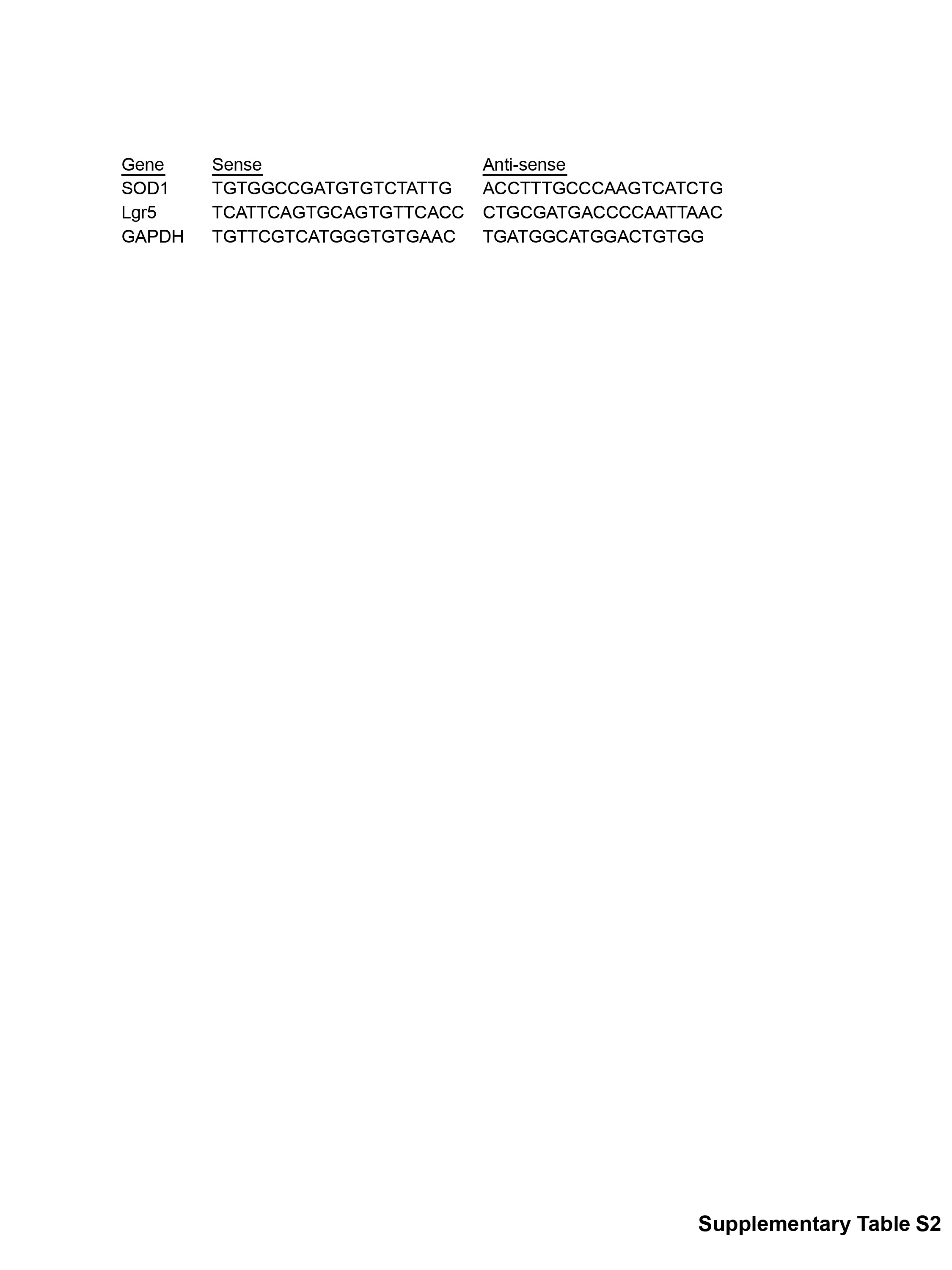

Supplement: S2 Table — (TIFF) [file pone.0211916.s011.tiff]
